# First record of Jacobsoniidae (Coleoptera) on the African continent in Holocene copal from Tanzania: biogeography since the Cretaceous

**DOI:** 10.1038/s41598-023-30368-7

**Published:** 2023-03-06

**Authors:** David Peris, Jörg U. Hammel, Chenyang Cai, Mónica M. Solórzano-Kraemer

**Affiliations:** 1 Institut Botànic de Barcelona (CSIC-Ajuntament de Barcelona), 08038 Barcelona, Spain; 2 Institute of Materials Physics, Helmholtz-Zentrum Hereon, Outstation at DESY, 22607 Geesthacht, Germany; 3 State Key Laboratory of Palaeobiology and Stratigraphy, Nanjing Institute of Geology and Palaeontology, and Center for Excellence in Life and Paleoenvironment, Chinese Academy of Sciences, 210008 Nanjing, China; 4 School of Earth Sciences, University of Bristol, Bristol, BS8 1TQ UK; 5 Department of Palaeontology and Historical Geology, Senckenberg Research Institute and Natural History Museum, 60325 Frankfurt Am Main, Germany

**Keywords:** Evolution, Zoology

## Abstract

Neither fossil nor living Jacobsoniidae are found in abundance. *Derolathrus cavernicolus* Peck, 2010 is recorded here preserved in Holocene copal from Tanzania with an age of 210 ± 30 BP years. This leads us to three interesting conclusions: (1) This is the first time the family was found on the African continent, extending the family’s distribution range to hitherto unknown localities. *Derolathrus cavernicolus* in Holocene copal from Tanzania expands the known distribution of the species, previously only recorded in the USA (Hawaii and Florida), Barbados, and Japan, both spatially and temporally. (2) All fossil specimens of the family have been found preserved in amber, which might be due to the small size of the specimens that prevents their discovery in other types of deposits. However, we here add a second aspect, namely the occurrence of this cryptic and currently scarce family of beetles in resinous environments, where they live in relationship with resin-producing trees. (3) The discovery of a new specimen from a family unknown on the African continent supports the relevance of these younger resins in preserving arthropods that lived in pre-Anthropocene times. Although we cannot demonstrate their extinction in the region, since it is possible that the family still survives in the already fragmented coastal forests of East Africa, we are detecting a loss of local biodiversity during the so-called Anthropocene, probably due to human activity.

## Introduction

**Figure 1:**
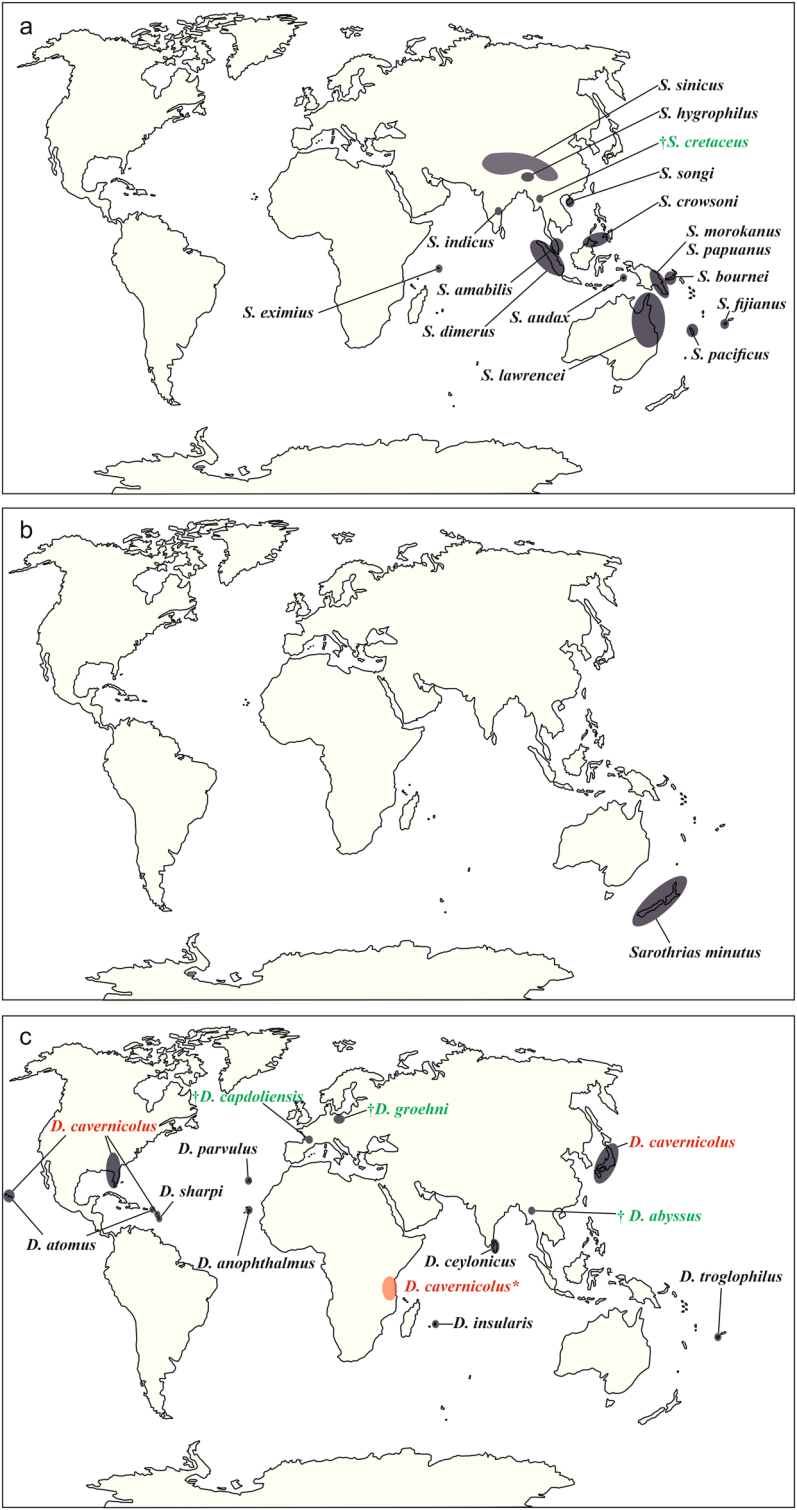
Distribution map of the Jacobsoniidae species with the fossil representatives indicated with †. The information is updated from Háva^2^ (**a**). *Sarothrias* spp.; (**b**). *Saphophagus* spp.; (**c**). *Derolathrus* spp., with the widespread distribution of *D. cavernicolus*. Fossil species are highlighted in green. *Derolathrus cavernicolus* indicated in red, with * denotes a probably extinction in the region.

Jacobsoniidae is a small family of polyphagan beetles, with only three extant genera—*Saphophagus* Sharp, 1886, *Derolathrus* Sharp, 1908 (in Sharp and Scott^1^), and *Sarothrias* Grouvelle, 1918—that account for 24 extant and four extinct described species^2–6^. All species in this family are extremely rare, occurring very sporadically in the Australian, Oriental, and Neotropical regions^2,7^ (Fig. 1).

All species in the family are less than 2.5 mm in length, which makes them difficult to detect, both in the wild and as fossils. In addition, little is known about their biology^7^. They are usually found in leaf litter and rotting wood. However, *Derolathrus* species have also been found in fungal fruiting bodies and bat guano^7^, and *Sarothrias* species are believed to be associated with ants^8^.

Due to the considerable morphological and behavioural differences between the three genera, it is not certain whether Jacobsoniidae, as presently defined, is in fact monophyletic. Historically, the family has been placed in different series of Coleoptera (see a review in Tihelka et al.^6^). The family was placed in the superfamily Dermestoidea together with Derodontidae, Nosodendridae, and Dermestidae^9^. Nevertheless, Crowson^10,11^ found similarities in morphological characters between Jacobsoniidae and staphylinoids, namely hind wing venation and maxillary galea in larvae and adults. The placement of Jacobsoniidae within Staphylinoidea as a sister group to a clade comprising Ptiliidae and Hydraenidae is strongly supported by recent phylogenomic studies^12–14^, although the Bayesian analysis based on eight gene markers suggests it is a sister group to Staphylinoidea^15^. The three very different-looking genera have not been included together in any recent analyses, and genetic data are only available for two of them: *Derolathrus*^14^ and *Saphophagus*^13^. Genetic data for the third genus, *Sarothrias,* would allow testing the family’s monophyly and determining a definitive placement of the family in Staphylinoidea.

Jacobsoniidae includes, at present, four extinct species, all of them described from different amber deposits (Fig. 1). *Derolathrus capdoliensis* Tihelka, Peris, Cai and Perrichot, 2022 was recently described as the oldest fossil of the family from the Albian-Cenomanian Charentese amber (France); it occurred almost contemporaneously with *D. abyssus* Yamamoto and Parker, 2017 and *Sarothrias cretaceus* Cai, Ślipiński, Leschen, Yin, Zhuo and Huang, 2017, both described from the Cenomanian Kachin amber (Myanmar)^4–6^. *Sarothrias cretaceus* was described based on two fossil specimens^4^. The sole fossil species out from the Cretaceous is *Derolathrus groehni* Cai, Leschen and Huang, 2016, described from two specimens preserved in the Eocene Baltic amber^16^. These fossils indicate that two out of the three current genera (i.e., *Derolathrus* and *Sarothrias*) have existed since the Cretaceous, representing examples of bradytely, or arrested evolution^17^. This long morphological stasis of diagnostic features in a fossil specimen, which makes it indistinguishable from current genera, has already been described in different fossil beetles from the Cretaceous^5,6,18,19^. In all cases, the small size of the specimens and a specific ecology could explain the stability of these lineages^18,20^.

The species *Derolathrus cavernicolus* Peck, 2010 has only been documented from Florida, Hawaii, Barbados, and Japan^21,22^ (Fig. 1c). We record it here preserved in Holocene copal (210 ± 30 BP years) from Tanzania. The Holocene copal and Defaunation resin of Tanzania were produced by *Hymenaea verrucosa* Gaertner, 1791, an angiosperm that today occurs only in certain parts of Eastern Madagascar and East Africa^23^. *Hymenaea verrucosa* is also closely related to *H. protera* Poinar, 1991, which is the originator of the Dominican amber, and to *H. mexicana* Poinar and Brown, 2002, and *H. allendis* Calvillo-Canadell, Cevallos-Ferriz and Rico-Arce, 2010, from which the Mexican amber originates^24^.

We used synchrotron-radiation microcomputed tomography (SRμCT) as a powerful tool for the study of inclusions in resins, favouring the detailed observation of this small specimen.

## Systematics

**Figure 2:**
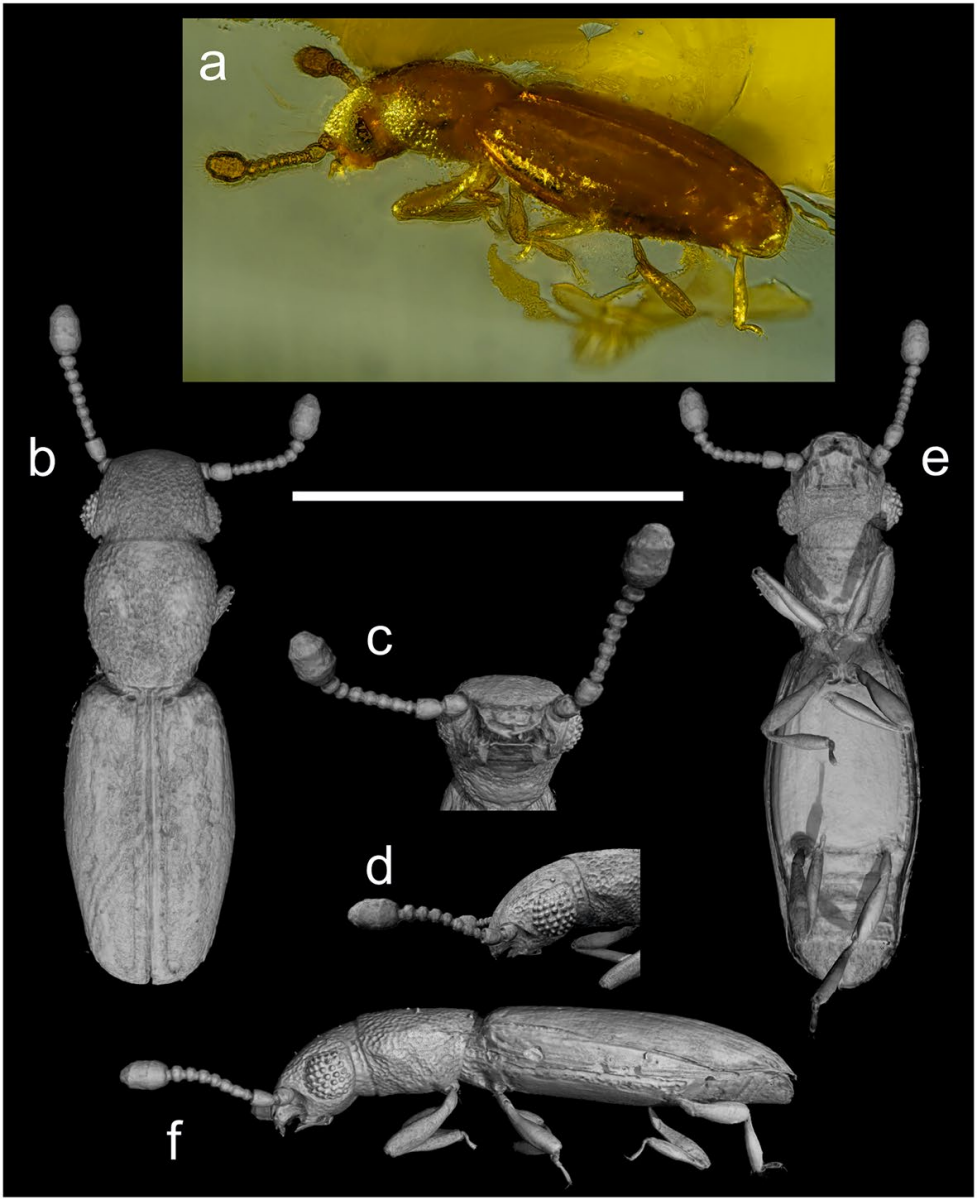
Specimen and virtual representation of *Derolathrus cavernicolus* in Tanzanian Holocene copal (210 ± 30 BP). Piece number SMF Be 3720.1a. (**a**) Specimen in dorso-lateral view. (**b**) Habitus in dorsal view. (**c**) Head in ventral view. (**d**) Head in lateral view. (**e**) Habitus in ventral view. (**f**) Habitus in lateral view. Scale bar 0.5 mm.

- Order Coleoptera Linnaeus, 1758
- Suborder Polyphaga Emery, 1886
- Superfamily Staphylinoidea Latreille, 1802
- Family Jacobsoniidae Heller, 1926
- Subfamily Derolathrinae Sen Gupta, 1979
- Genus *Derolathrus* Sharp, 1908
- *Type species*. *Derolathrus atomus* Sharp, 1908
- *Derolathrus cavernicolus* Peck, 2010
- Figure 2

*Material* SMF Be 3720.1a, preserved in Tanzanian Holocene copal and housed in the amber collection at the Senckenberg Research Institute and Natural History Museum Frankfurt, Germany. Syninclusions: a Coleoptera: Curculionidae, a Lepidoptera, a Diptera: Cecidomyiidae, and a Hemiptera: Sternorrhyncha.

*Remarks* The fossil beetle is easily placed in Jacobsoniidae based on its distinctive small size, elongate body shape, the greatly elongated metathorax, and its short abdomen^7^. The specimen can be placed in *Derolathrus* based on the following combination of characters: body minute (less than 1 mm in length) and narrowly elongate, prothorax elongate and posteriorly narrower, mesoscutellum not visible, tarsal formula 3–3–3, and metaventrite longer than all five visible abdominal ventrites combined^21^. The new specimen is placed in *Derolathrus cavernicolus* using the key to *Derolathrus* species provided in Yoshitomi and Hisasue^22^. A series of characters made it possible to differentiate this species from other congeners, i.e., eyes large, their diameter greater than the distance from their anterior margin to base of antennae; pronotum slightly longer than wide; dorso-lateral edge without carina; presence of a dorsal, longitudinal “Y”-shaped or subtriangular depression, which is widest at its anterior end; elytra entire, covering all of the abdominal tergites; elytral striae present only as complete sutural stria and partly developed second stria; sutural stria originating from pit near inner apical margin^21,22^. The diagnostic character of large setiferous punctures dorsally in head and pronotum^21^ is not observed in the illustrations of the specimen from Japan^22^, nor in the fossil specimen here. This feature is of rather medium size in the fossil specimen (Fig. 2), and small in the specimen from Japan. The original description of the species also pointed out that the head was slightly narrower than the widest part of the prothorax^21^. However, this character was not observed in the specimen from Japan, which shows a head as wide as the prothorax^22^, nor in the fossil specimen, which shows a head slightly wider than prothorax (Fig. 2b). The antennomere three is more robust in the description of Recent fauna, appearing to be 0.8 times the width of the pedicel^21,22^, while it is more delicate in the fossil specimen, at 0.6 times the width of the pedicel (Fig. 2b,c,e). Finally, the surface of the elytra is smooth in the original description, but the specimens from Japan and the fossil specimen described here show sparsely punctured elytral surfaces, following the elytral striae (^22^ and Fig. 2b). However, the differences described here are not distinctive enough and at times even intermediate among the specimens; therefore, they do not support the description of new species based on the available specimens.

*Biological notes* The specimens of this species were collected from leaf litter in Japan and from bat guano in caves in Florida and Barbados^21,22^.

## Discussion

### Distribution of Jacobsoniidae

Extant members of the family Jacobsoniidae are distributed worldwide, although they are particularly well represented in tropical areas and on oceanic islands (Fig. 1). Interestingly, they are not known from the South American landmass. Moreover, Europe and the African landmass are, until today, only represented by inclusions in Cretaceous and Eocene amber and in Holocene copal, respectively. Of the 24 current species in the family, 18 are found in isolated countries such as Papua New Guinea, New Zealand, or New Caledonia. On the continental mainland, only *Sarothrias hygrophilus* Pal 1998 and *S. indicus* Dajoz, 1978 are found in India, *S. lawrencei* Löbl and Burckhardt, 1988 in Australia, *S. sinicus* Bi and Chen, 2015 and *S. songi* Yin and Bi, 2018 in China, and *Derolathrus cavernicolus* in Florida (USA)^2^. *Saphophagus* is only know from the monotypic species *S. minutus* Sharp, 1886 restricted to New Zealand^2^. Only two species in the family show a wider distribution compared with other species in the family. *Derolathrus atomus* is reported from the Hawaii islands and the West Indies^2^, and *Derolathrus cavernicolus* represents the most widespread species in the family, reported from the USA (Hawaii and Florida), Barbados, Japan, and now in Holocene copal from Tanzania (Fig. 1c).

How such small beetles can show a worldwide, yet highly endemic distribution is an intriguing question. Their particular distribution is understood as the result of long-distance dispersal, which is a natural dispersal mechanism in the family. It is facilitated by air currents, aided by the beetles’ feather-like hind wings and small body size^16,21^. Since some species appear to feed on fungi in soil and would naturally occur in association with soil on plant roots and in leaf litter, it has been suggested that their distribution pattern may be associated with anthropogenic causes, such as navigation or the introduction of plant species from many parts of the tropical world^21^. However, this explanation remains unsatisfactory and raises the question of why their long-distance dispersal by means of natural or anthropogenic mechanisms would be restricted to islands or isolated land areas rather than entire continents. On the other hand, it is possible that the beetles had a much wider distribution in the past, and the current endemism is due to regional extinctions, combined with new colonisations^6,16^. A divergence time analysis, calibrated using fossil data, revealed an Early Jurassic origin of Jacobsoniidae, at which time they split from their sister families Ptiliidae and Hydraenidae^14^. The land distribution at that time indicates that the family was already widespread when the supercontinent Pangea began to separate significantly for the first time. The fossil record of the family encompasses the Tethyan and Austral realms in the Cretaceous, and the Afrotropical realm in the Holocene. This fossil distribution suggests that Jacobsoniidae is a very ancient group, and that the group was more diverse in the past than it is in the present^4^. It appears that the family currently has a limited distribution, as it was unknown from the African continent until the description of the specimen here. The geographically closest relative is the species *D. anophthalmus*, which has been reported from the Canary Islands. However, Cai et al.^16^ cited unstudied material and undescribed species from Africa, Australia, the Neotropics, New Caledonia, and the Solomon Islands. Such new information would help to understand the distribution of this exceptional family and complement the conclusions obtained here.

### Ecology of Jacobsoniidae

There are now four fossil species known in Jacobsoniidae, all of them described from amber inclusions: one species from the Albian of France^6^, two species from the Cenomanian of Myanmar^4,5^, and one species from the Eocene of Poland^16^. All of these organisms were found in ambers of different gymnosperm origin. The specimen identified here as *Derolathrus cavernicolus* is preserved in Holocene copal from Tanzania, which has an angiosperm origin. It is the fifth species preserved in resin of a different maturity, age, and botanical origin.

The answer to the question why all of the known fossil specimens in the family were found in amber or copal deposits may be found in the fossilisation process. In this regard, the characteristics of the resins themselves, the small size of this type of beetles, and what we consider the most relevant aspect, their biology, played an essential role. Many of the specimens preserved in ambers are small. Historically, it was thought that arthropod fossilisation in amber was biased with regard to body size. However, the size distribution of arthropods preserved in diverse ambers is similar to the general body size distribution of living insects in similar environments, and the size bias is qualitatively independent of the kind of trap for non-extreme values^25^. For organisms such as spiders or ants, it has been demonstrated that the size of specimens enclosed in amber depends more on the complexity of the forest structure and the biology of the organism rather than resin entrapment-related biases^26,27^. More specifically, this means that selected taxa trapped in resins represent the fauna living in and around the resin-producing trees and appear in resins because of their ecology and behaviour, usually closely related to a tree-inhabiting life^27^. Still, without the size bias, the high-quality preservation of specimens in amber offers a rich record of fragile and hard-to-preserve fossils. This includes specimens that would otherwise go unnoticed in compression deposits, since it is challenging to detect fossils of such a small size. Nevertheless, it also implies that species of Jacobsoniidae had some degree of relationship with the resin-producing plants, both gymnosperms and angiosperms, since the Cretaceous.

The biology of jacobsoniids is poorly known. They have been collected from leaf litter and rotten wood^7^, but they are also associated with fungi and bat guano^28^. *Sarothrias* may be myrmecophilous based on an overall morphology that possibly suggests an inquilinous lifestyle^28^, and several specimens of *S. lawrencei* were collected from ants’ nests (H. Escalona, pers. obs. in^4^). The morphology of the group appears to have changed little since the Cretaceous^5,6^. Moreover, this morphological stasis may result from a stabile microhabitat throughout geological times, providing ‘refuge niches’^29,30^. Thus, some taxa are protected from strong selective pressures and extinction, something similarly described in different beetle species before^5,18^.

### Biodiversity loss

Amber is a fossil material well known for its capacity to preserve biological inclusions from ancient ecosystems with remarkable fidelity^31^. The organisms preserved in copal and Defaunation resin are also of scientific interest^32,33^, because these samples document the impact, principally on arthropod biodiversity, of Recent climatic events and ecosystem changes on a shorter timescale, such as anthropogenic effects^33^. Nature is declining globally at unprecedented rates in human history and this loss is a direct result of human activity^34^. As a result, modern species no longer occur in a geographic area because the destruction of their habitat is directly or indirectly caused by human activity. In this regard, endemic species or those with a very narrow niche are particularly affected. The small family of jacobsoniid beetles, particularly found on tropical oceanic islands (Fig. 1), is unknow from the African continent, with the only exception of the specimen of *Derolathrus cavernicolus* from the Tanzanian Holocene copal described here (Fig. 1c). With a more widespread distribution of the species on other continents, *D. cavernicolus* may yet be undiscovered on the African continent, or it may already be one more example of extinction in the region.

Many modern species are well known from copal and Defaunation resin, and some species were first documented in such resins and were later discovered to be living in the same environs today. However, other species from copal and Defaunation resin remain the only known representatives of their taxon (see examples in Solórzano-Kraemer et al.^33^). Coastal forests of Eastern Africa are ranked among the world’s priority biodiversity hotspots^35^, with a high incidence of forest-obligate endemism or threatened species with narrow geographic ranges that are often endemic to a single site or forest patch^36^. However, at the same time it is a region of increased anthropogenic impact, inducing climate change and loss of biodiversity, which is projected to increase in magnitude^37^. Thus, it is likely that species in copal and resin from this region have recently become extinct due to human activity^33^. This emphasises the importance of investigating copal and Defaunation resin biotas as a source of data for exploring recent impacts of habitat and climate change. To confidently assess that *D. cavernicolus* is a recently extinct species from this region, and not a species present in the modern African fauna albeit as yet unrecognised, extensive efforts to search for individuals of this taxon should be made in these still preserved coastal forests. However, the absence of the family from some of the large landmasses on Earth (South America, Europe, and Africa) and its almost complete absence from North America, together with the highly endemic distribution of the different species, suggests that the regional extinction hypothesis is the most likely. In fact, a more widespread distribution of jacobsoniid beetles during the Cretaceous in Europe and Asia is suggested based on amber^4,6^. Despite their more or less widespread distribution among islands worldwide^2^ and their facility to spread via air currents^16^, it has been suggested that, at least to some extent, their current range is the result of anthropogenic introductions^21^. Moreover, the current biogeographical range of Jacobsoniidae may be the remnant of a more widespread distribution in the past due to extinction in large regions on Earth, with the main refugia on oceanic islands in tropical areas. The analysis of the reasons for such diversity loss and limitation of geographic distribution is a line of research that could be developed in future publications.

## Material and methods

### Material

One specimen from the beetle family Jacobsoniidae has been studied and identified as *Derolathrus cavernicolus* (SMF Be 3720.1a) in Holocene copal from Tanzania. The copal and Defaunation resin collection was donated to the Senckenberg Research Institute and Natural History Museum Frankfurt between the years 1874 and 1901 and was labelled as “Madagascar or Zanzibar copal”. However, the included fauna suggests an origin on the African continent and not in Madagascar^33^. The material is housed in the amber collection at the Senckenberg Research Institute and Natural History Museum Frankfurt, Germany (SMF).

Copal and Defaunation resin from Tanzania come from the coastal vichaka forests (vichaka meaning “scrub” in Swahili), unique areas of dry lowland evergreen forest, woodland, and particularly bushland interposed between the Indian Ocean littoral mangroves and the Eastern Arc rainforests situated approximately 150 km inland (Fig. 1 in Solórzano-Kraemer et al.^33^). During the nineteenth century, copal and Defaunation resin from the Zarano region, from Bagamoyo in the north to Lindi in the south, were intensively traded, with 95% of the East African copal originating from this area^38^.

### Preparation and radiocarbon analyses

The piece was cut and polished at the SMF using a Phoenix Beta polishing machine with grinding paper for metallography wet and dry: Grip 1200, 2500, and 4000. After being polished, it was stored following Sadowski’s et al.^39^ recommendations.

The age was determined using rigorous standard methods of radiocarbon analysis (^14^C and ^13^C) in the Beta Analytic Inc. Laboratories (www.radiocarbon.com/), prior to scanning with synchrotron-radiation based X-ray microtomography (SRμCT). The results suggest the specimen’s age as 210 ± 30 BP. With a 60.3% probability, the resin was produced between the years 1719–1830 cal AD, as indicated by the calibrated calendar year (cal AD). The Beta Analytic results also indicate, with a 24.1% probability, an age of 1651–1711 cal AD. BP means conventional radiocarbon age, and “Present” is defined as 1950 AD. We follow Solórzano-Kraemer et al.^32^ for the classification of copal and resin: Defaunation resin is younger than 1760 AD, and the Holocene copal is 0.0117 Ma–1760 AD. This specimen corresponds to the limit of the Holocene and can be classified as Holocene copal.

### SRμCT

The specimen was analysed using synchrotron-radiation based X-ray microtomography. The imaging was performed at the Imaging Beamline—IBL P05—PETRA III at Deutsches Elektronen Synchrotron (DESY) in Hamburg, operated by the Helmholtz-Zentrum Hereon^40,41^. 3601 projections, equally spaced between 0 and π, were recorded for a tomographic scan at a photon energy of 18 keV. A custom-build 20 MP CMOS detector^42^ with an effective pixel size of 0.64 µm and a sample-to-detector distance of 30 mm was used to record the images. Tomographic reconstruction was achieved by applying a transport-of-intensity phase retrieval with a beamline-specific workflow^43^ implemented in Matlab (Math Works), using the filtered-back projection algorithm implemented in the Astra Toolbox^44–46^. Raw projections have been binned two times for processing, resulting in an effective pixel size of 1.28 µm in the reconstructed tomographic volume.

The specimens were segmented in three dimensions using region-growing techniques in VGStudioMax (version 3.3.1 www.volumegraphics.com/de, Volume Graphics, Heidelberg, Germany).

### Imaging

The micrographs and Z-stack images were recorded under a compound microscope Olympus CX41 equipped with a digital camera sCMEX-20. These micrographs were processed with Image Focus Alpha, version 1.3.7.12967.20180920 (www.euromex.com), and finally merged with Combine ZP, version 1.0 (www.combinezp.software.informer.com).

Figures were created using CorelDRAW Graphics Suite software, version 19.0 (www.coreldraw.com).

## Data Availability

All data required to evaluate the conclusions offered here are contained within the paper. Requests for data related to the material in the paper such as SRµ-CT scans may be directed to Mónica M. Solórzano Kraemer (monica.solorzano-kraemer@senckenberg.de).
